# The Impact of Stress on the Functioning and Survival of Transplanted Kidneys—Biological Mechanisms, Clinical Implications, and Therapeutic Perspectives

**DOI:** 10.3390/ijms262412041

**Published:** 2025-12-14

**Authors:** Paulina Piechowiak, Katarzyna Aleksandra Lisowska

**Affiliations:** 1 Division of Clinical Psychology, Faculty of Health Sciences with the Institute of Maritime and Tropical Medicine, Medical University of Gdańsk, 80-210 Gdańsk, Poland; paulina_piechowiak@gumed.edu.pl; 2 Department of Rheumatology, Clinical Immunology, Geriatrics and Internal Medicine, Faculty of Medicine, Medical University of Gdańsk, 80-214 Gdańsk, Poland

**Keywords:** stress, kidney transplant, end-stage renal disease, cytokines, cortisol, ACTH

## Abstract

Stress is well known to affect the immune system. However, in patients suffering from end-stage renal disease (ESRD) who underwent a kidney transplant (KT), the problem of stress appears to still be underestimated. The review aims to discuss the influence of chronic stress on the immune function and the relationship between chronic stress and the fate of a transplanted kidney. Therefore, we analyzed the relevant literature, searching for articles linking chronic stress to kidney transplant. Although the biology of stress is well documented in the literature, its impact on the fate of transplanted kidneys remains poorly understood. We have found only a limited number of studies examining the role of stress in patients after kidney transplantation, especially regarding graft function. However, a few studies have shown that psychological and behavioral interventions appear to reduce perceived stress and improve quality of life among KT patients. Here, we present the Integrated Stress–Immune–Transplant Kidney Model that highlights how stress-related neuroendocrine signals converge with immune and vascular processes to shape chronic immune activation and, ultimately, graft outcomes. It also incorporates behavioral and psychological responses as downstream modulators of the same pathway, rather than parallel or unrelated factors. Addressing this problem in transplant rejection requires an integrative biopsychosocial approach that combines psychological assessment, biological monitoring, and supportive interventions as part of routine post-transplant care.

## 1. Introduction

In Poland, there are currently around 2000 people waiting for a transplant, the largest group of whom are patients who need a kidney transplant (KT). KT is the treatment of choice for patients with chronic kidney disease (CKD) in end-stage renal disease (ESRD) because it is associated with longer survival and better quality of life compared to dialysis therapy. However, immunological reactions directed against the donor kidney can lead to acute or chronic rejection of the transplant.

The significant development of transplant medicine over the last few decades has contributed to a substantial reduction in the percentage of patients who reject a transplanted kidney. In the 1980s, this problem affected as many as 50–60% of KT recipients [1]. Currently, this problem affects up to 20% of KT patients [2]. The rate of acute rejection in the first year after transplantation is 1–2% lower with living donor transplantation compared with deceased donor transplantation. Acute (AR) and chronic rejection (CR) can be antibody-mediated (ABMR) or T-cell-mediated (TCMR). Acute rejection of the transplanted organ predisposes to chronic graft dysfunction. Data on the incidence of TCMR and ABMR are from centers that perform surveillance or protocol biopsies, and as such, may differ from the estimates provided above. In a meta-analysis of 12 studies on patients receiving tacrolimus and mycophenolate, the incidence of acute TCMR was 16% within the first year after transplantation [3]. In another systematic review, the prevalence of acute ABMR ranged from 1.1 to 21.5% [4]. The prevalence of chronic ABMR ranged from 7.5 to 20.1% over 10 years of follow-up. Regardless of whether graft rejection is mediated by antibodies or T cells, these processes are regulated by a network of cytokines and cytokine receptors on the surface of immune cells [5,6].

Chronic illness is one of the most severe stressors in a person’s life, and organ transplantation can be characterized as a crisis for the patient, in terms of its breakthrough nature and the need to adapt to a new situation, a highly stressful event. The population of transplant patients is a significant group due to its crucial role in the process of transplant rejection and the occurrence of inflammatory conditions. Although the development of transplant medicine over the past several decades has contributed to a reduction in the percentage of patients experiencing rejection of a transplanted kidney, this issue currently affects a maximum of 20% of KT recipients [2], which still means that one in five patients after transplantation will experience organ rejection. A network of cytokines and their receptors on the surface of immune cells regulates transplant rejection. On the other hand, these cytokines and chemokines secreted by immune system cells are balanced with hormones and neurotransmitters that regulate the stress response.

While multiple reviews have separately addressed the impact of psychological stress on immune function or graft outcomes, an integrative framework linking neuroendocrine stress pathways, immune dysregulation, endothelial injury, and patient behavioral factors in kidney transplantation remains lacking. Existing literature typically examines these dimensions in isolation—for example, focusing on hypothalamic–pituitary–adrenal (HPA) axis abnormalities, cytokine activity, adherence-related behaviors, or psychosocial vulnerability—but not their interactions. The present review aims to fill this gap by proposing the Integrated Stress–Immune–Transplant Kidney Model, a conceptual framework that synthesizes psychological, biological, and behavioral mechanisms into a unified explanatory pathway. This model illustrates how stress-induced neuroendocrine changes propagate through immune and endothelial systems, shape immune activity, and influence graft outcomes, while also linking these processes to patient coping, adherence, and resilience. By structuring the available evidence within this multidimensional model, the review offers a coherent perspective that has not been articulated in previous publications.

## 2. The Importance of Stress in Transplant Medicine

The concept of stress emerged, quite perversely, from the scientific community’s interest in the body’s well-being. Claude Bernard laid the foundation for the study of homeostasis with his research on the body’s ability to maintain internal balance, and Walter Cannon, the creator of the concept of the fight-or-flight response, continued his work, focusing on the mechanisms that maintain homeostasis [7]. Until this point in history, stress was a physiological disruption of homeostasis, a topic of lesser interest than how the body coped with it and returned to it. Cannon himself rarely used the term “stress” and never defined it. It was Hans Selye [8] who focused on the phenomenon of stress and created its first definition, as “physiological adaptive responses to perceived (psychological) or real (physical) threats (“stressors”) to an organism” [9].

As a species, humans are facing a very interesting situation regarding stress responses. On the one hand, stress (and its associated physiological processes) does not require consciousness. For example, in patients under deep sedation, significant stimulation of the adrenal medulla continues to occur unconsciously in response to acute glucoprivation. Stress (and its associated physiological processes) do not require the actual presence of a stimulus—simply thinking about it is enough to disrupt homeostasis and initiate the stress response [10]. This allows humans to experience chronic stress through rumination, anticipation, and even imagining difficulties and challenges. Furthermore, individuals not only anticipate stress on a psychological level (thoughts and imaginations) but also assess their ability to cope with it. In this configuration, distress (a form of stress) may occur in an individual, not so much in response to the triggering stimulus (physical or mental) as to the belief that they lack sufficient resources to cope with the challenge. Research has shown that the body experiences distress when it recognizes that the efforts required to reduce stress will be extremely resource-intensive or even beyond the system’s capabilities [11].

The complexity of stress poses an intriguing challenge when determining its level, requiring an interdisciplinary approach that draws on methods from both medicine and psychology. From a medical perspective, cortisol levels in blood or saliva are commonly measured, with support from other indicators such as a complete blood count and lipid profile. From a psychological perspective, the most used measures include the Perceived Stress Scale (PSS), the Perceived Stress Questionnaire (PSQ), and the Depression, Anxiety, and Stress Scale-21 Items (DASS-21), depending on the needs of the study.

Given the current known negative impact of stress on a healthy body and the pathogenic potential of particularly chronic stress and distress, it seems logical to assume that their effect on the functioning of individuals coping with illnesses or psychophysical disorders will be even more severe. Research on the relationship between stress, disease, and treatment processes currently encompasses virtually all human ailments. Stress is being studied for most, if not all, diseases—in gastroenterology in connection with its supposed impact on irritable bowel syndrome [9], in cardiology as one of the variables influencing cardiovascular disease [12], in oncology, investigating how stress contributes to cancer [13], in endocrinology, which sees stress as an extremely important factor in the exacerbation of polycystic ovary syndrome (PCOS) symptoms [14] and, of course, in psychiatry, where a strong negative impact of stress on mental functioning is observed not only in individuals with pre-existing psychiatric conditions but also in mentally healthy individuals experiencing excessive stress [15]. Stress, however, appears to be a particularly important variable in the context of illnesses and disorders that directly resonate with the physiological aspects of stress.

There is a strong correlation between some inflammatory markers and the neuroendocrine system in chronic stress [16]. This relates to the network of cytokines and neurotransmitters, along with their receptors. Neurons and glial cells can secrete cytokines within the central nervous system (CNS). On the other hand, those secreted by lymphocytes, monocytes, or macrophages, e.g., interleukin 6 (IL-6), can penetrate the blood–brain barrier (BBB) [17]. Chronic stress often leads to elevated levels of pro-inflammatory cytokines, such as IL-1, IL-6, and TNF-α (tumor necrosis factor alpha). In KT recipients, serum IL-6 and TNF-α levels often are elevated and correlate significantly with kidney transplant rejection [18]. Therefore, it should come as no surprise that researchers are interested in stress in the context of patients whose lives may be threatened by inflammation, and who simultaneously experience one of the most severe stressors for transplant patients—a chronic illness and a life-threatening condition.

## 3. Psychoneuroimmunology as an Integrative Perspective in Transplantation

It is now known that psychological stress triggers a series of physiological processes and, in cases of high intensity or chronic occurrence, can lead to a situation referred to as “allostatic overload” in an individual [19]. The fact that our physiology does not differentiate between physical and psychological stimuli and responds with the same physiological mechanisms compels us to pay attention to more than just the environment and to look “inside” the patient.

The term psychoneuroimmunology was first used in 1980 by Robert Ader in a lecture to the American Psychosomatic Society [20]. In his lecture, Ader drew attention to research indicating that the joint actions of all systems in the human body are aimed at maintaining health. Psychoneuroimmunology is therefore a field that integrates what has often been considered separately until now (perhaps due to the prevailing Cartesian dualism) and aims to understand the relationship between mental functioning and the human immune system, and vice versa. In research trends, there is particular interest within psychoneuroimmunology not only in the impact of psychopathologies, such as depression or anxiety disorders, but also in the connections between various forms of stress and the holistically understood health status of the patient [21].

Psychoneuroimmunology describes the bidirectional relationships between the CNS and the immune system and their involvement in the occurrence of a range of psychological and neurological symptoms. One commonly studied hypothesis posits that a persistent immune response and ongoing chronic inflammation influence changes in cognitive, behavioral, and emotional functioning in patients, and may even contribute to the development of depressive disorders [22]. Assuming that the relationships between systems in the psychoneuroimmunological model are bidirectional, this suggests an interesting hypothesis regarding the potential influence of the patient’s psychological functioning on the operation of their immune system and the occurrence of inflammatory states.

Interestingly, the relationship between stress and immune function was observed many years before studies in psychoneuroimmunology were established. The long-term expansion of understanding of the concepts of stress and “coping with stress,” as well as their linkage with stable personality traits, has led to the perspective of psychoneuroimmunology that we use today. It is recognized that personality traits and coping styles, as part of individual differences, can influence an individual’s immune functioning [21]. In view of the role of psychological stress, it is time to take into account not only the environment and the stressors coming from it, but also the patient themselves—their personality structures (and consequently their worldview, cognitive functioning, social relationships, and ability to regulate emotions) as well as their capacity to cope with experienced stress (both its psychological challenges and physiological components).

However, given the multitude of variables, more research is needed to define the extent of this influence clearly and perhaps to learn to manipulate it sufficiently so that it could serve as a tool to support treatment, especially for chronic diseases.

Given the close relationship between inflammatory states and organ rejection, psychoneuroimmunology at first glance seems an ideal partner for collaboration with transplantology. However, due to the extraordinary complexity of the immune system, attempts to construct models of transplant psychoneuroimmunology still remain merely experimental. Particular difficulties arise from the enormous number of variables that each system “invites” to participate in hypothesis formation. There is a need for numerous studies to verify as many specifically narrowed and operationalized hypotheses as possible. Without this, our understanding of how disturbances in mental functioning affect immunological function in transplant patients will remain rudimentary [23].

## 4. Mechanisms Through Which Stress Could Affect the Fate of a Transplanted Kidney

The nervous system can regulate the function of lymphatic organs and immune system cells through hormones, secreted mainly by the pituitary gland, and neurotransmitters, such as noradrenaline, released directly from the axons of cells of the autonomic nervous system [24,25]. Lymphocytes, monocytes, and macrophages are also known to express on their surface receptors for several neurotransmitters (e.g., noradrenaline, acetylcholine, dopamine, serotonin) [26,27], and hormones (e.g., ACTH, GCs) [28,29], allowing them to respond to signals sent by the nervous system [28]. In response to neurotransmitter or hormone stimulation, immune cells are activated and begin to produce cytokines and hormones, including ACTH, triiodothyronine, endorphins [30], and serotonin [31].

Psychological and physiological stressors can profoundly influence immune and metabolic homeostasis, thereby affecting the survival and function of transplanted organs. In KT recipients, chronic activation of neuroendocrine and inflammatory pathways induced by stress may contribute to both acute and chronic graft injury [32]. These effects arise through a combination of direct biological mechanisms associated with a sustained proinflammatory milieu that undermines graft survival, and indirect behavioral mediators that compromise adherence and metabolic stability [33].

### 4.1. Activation of the HPA Axis in Chronic Stress

The neuroendocrine system mediates immunologic reactions to stress stimuli. The physiological role of acute mental stress is to enhance immune function [34]. Acute mental stress stimulates a catecholamine-mediated increase in white blood cell count [35,36], thereby enhancing the nonspecific innate immune response [37]. It leads to rapid activation and trafficking of neutrophils and monocytes into the injury site [38]. Catecholamines (CAs) may also cause cytokine release from natural killer cells (NK cells) [39,40]. Acute mental stress may also enhance the adaptive immune response, T-cell- and B-cell-dependent [41]. It also activates the HPA axis, leading to the release of GCs from the adrenal cortex. The role of GCs in an acute stress situation is to silence the inflammatory response by reducing the production of pro-inflammatory cytokines and increasing the production of anti-inflammatory cytokines, which is associated with the activation of anti-inflammatory M2 macrophages [42]. GCs also activate macrophages to phagocyte apoptotic cells and tissue debris during inflammation resolution [43].

While high GCs are adaptive in acute settings, chronic stress maintains persistently elevated cortisol and catecholamine levels, which dysregulate immune signaling and promote inflammation. Prolonged HPA activation may impair GR sensitivity, paradoxically leading to steroid resistance and enhanced proinflammatory cytokine expression—a phenomenon well documented in stress-related disorders and increasingly recognized in the transplant population.

Under chronic stress, cortisol is continuously released in response to ACTH produced in the pituitary. Chronically elevated cortisol levels in the blood lead to downregulation of glucocorticoid receptors (GR) on inflammatory immune cells [44]. When cortisol fails to downmodulate inflammatory cell activity, we are dealing with a condition known as cortisol or steroid resistance (SR) [45]. The hypothalamus and pituitary also lose their sensitivity to GCs, which sustain production of CRH and ACTH [46]. ACTH secreted by the pituitary gland further stimulates the adrenal glands to produce cortisol [47], which eventually leads to adrenal exhaustion and a decrease in cortisol production [48]. The resistance of immune cells to GCs results in increased production of pro-inflammatory cytokines IL-1, IL-6, and TNF-α [49] with decreased IL-10 production [50,51]. In transplant recipients, such cytokine patterns overlap with molecular signatures of graft rejection. Studies show that serum levels of IL-6 and TNF-α are significantly higher in patients who rejected KT compared with patients with stable transplant [18].

Studies on cortisol levels in patients with ESRD yield conflicting results [52]. Serum or plasma cortisol levels were measured in some studies in CKD patients in the morning [53,54,55], whereas in others in the evening or even late at night [56]. Some authors reported high serum levels [53,56] or no difference compared with healthy individuals [55]. N’Gankam et al. [53] demonstrated that ESRD patients have elevated plasma cortisol and its metabolites in the morning, which are not adequately removed by dialysis. Raff and Trivedi [56] observed that ESRD patients exhibit elevated late-night plasma cortisol and ACTH levels, suggesting a disturbed circadian rhythm. Vigna et al. [54] found that basal serum cortisol and ACTH levels in ESRD patients were at the upper limit of the normal range, but the mean values did not differ from those in healthy people. Clodi et al. [55] showed that even in patients with normal cortisol levels, serum ACTH is elevated, suggesting activation of the HPA loop to maintain normal cortisol levels.

In KT patients, long-term glucocorticoid therapy has been a key component of immunosuppression for decades. GCs are also used as a regimen for acute rejection of the transplanted organ [57]. However, they have many side effects, which are associated not only with the risk of bacterial, fungal, and viral infections, obesity, metabolic syndrome, and adrenal deficiency but also with neuropsychiatric disorders, including mood changes and confusion, sleep disorders, and anxiety [58]. These negative effects of using GCs can be amplified by chronic stress, which will also contribute to a further increase in steroids. Unfortunately, because KT patients receive GC as part of immunosuppressive therapy, cortisol levels cannot be used to assess stress levels in this patient group.

### 4.2. Dysregulation of Immune Response

Chronic stress also alters adaptive immune function. Over time, immune cells, in the presence of high cortisol, decrease their responsiveness to cortisol, among other mechanisms, by decreasing GR expression or changing its sensitivity to GCs [59]. Altered glucocorticoid signaling can shift the immune balance toward Th17 dominance [60]. Regarding Tregs, studies yield conflicting results. According to Seissler et al. [61], a methylprednisolone bolus increases the percentage of activated Tregs during the first few days after kidney transplantation. However, in transplantation, the most important factor is the Th17/Treg balance, because the dominance of Th17 over Tregs can lead to acute rejection in the first few weeks after transplantation [62,63]. A study by Ma et al. [64] showed that KT recipients have higher Th17 cell numbers than before transplantation, which could disrupt the Th17/Treg ratio even when Treg numbers are higher.

### 4.3. Endothelial Dysfunction and Microvascular Injury

Kidneys are highly vascular organs receiving approximately 20% of cardiac output, and endothelial dysfunction and microvascular inflammation are among the most important reasons for graft dysfunction and rejection after kidney transplantation [65]. Studies have shown that endothelial cells (ECs) are an important target of GCs under physiological conditions [66]. GCs exert anti-inflammatory action towards ECs by inhibiting their expression of VCAM-1 [67,68] and ICAM-1 [68]. GCs also stimulate endothelial nitric oxide synthase (eNOS) activity, leading to increased nitric oxide (NO) production and vasodilation [69]. However, in a state of chronic stress, neuroendocrine activation affects endothelial integrity through oxidative stress and altered NO bioavailability [70,71]. It is associated with proinflammatory, pro-oxidative, and prothrombotic activity of ECs. ECs exposed to high GC levels for a prolonged period exhibit reduced NO production, which promotes vasoconstriction [72]. Moreover, it has been demonstrated that CRH, which is responsible for activation of the HPA axis, can stimulate monocyte adhesion to endothelium [73]. It also stimulates monocytes to produce proinflammatory cytokines that damage the endothelium [74]. High levels of GCs are also associated with hyperglycemia, which not only impairs NO production but also contributes to oxidative stress and vascular inflammation [75]. Persistent microvascular dysfunction may thus serve as an early link between psychological stress and chronic allograft nephropathy.

### 4.4. Behavioral and Metabolic Mediators

Beyond biological mechanisms, stress indirectly affects transplant outcomes through behavioral and metabolic pathways. One of the problems is non-adherence to immunosuppressive medications, which is very high among kidney transplant patients [76]. Alongside demographic factors, psychological distress is a well-established risk factor for nonadherence to immunosuppressive therapy, which in turn increases the likelihood of acute [77] and chronic [78] graft rejection. Achille et al. [79] pointed out that while 85% of KT patients reported never forgetting their immunosuppressants, only 62% took them exactly as prescribed. The authors using the Perceived Stress Scale (PSS), the transplant-related stress scale, and a French version of the ‘Psychological Distress’ subscale of the Psychosocial Adjustment to Illness Scale Self-Report (PAIS-SR) showed that patients admitting less than perfect adherence also experienced more stress in their everyday life in general than those who reported perfect adherence. Studies also show that KT recipients with more post-transplant complications (e.g., infections) were more likely to exhibit higher rates of non-adherence [80].

Additionally, several stress-related factors, including alterations in sleep, insulin resistance, hypertension, and dyslipidemia, were identified as determinants of allograft dysfunction. For example, Prather et al. [81] showed that poor sleep after lung transplant was a risk factor for chronic allograft dysfunction. Chronic stress is also associated with insulin resistance [82], which is recognized as a cause of chronic renal transplant dysfunction [83]. Another consequence of stress is lipid disorders, as demonstrated by Assadi [84]. The author proved that people experiencing mild psychological stress were more likely to develop dyslipidemia. Recent findings show that dyslipidemia is associated with a higher risk of chronic allograft vasculopathy [85]. It is most likely related to excessive activation of immune cells, especially T and B cells [86]. Cholesterol can directly bind to the β chain of the T-cell receptor (TCR), facilitating TCR clustering on the T-cell surface and increasing the TCR’s avidity for foreign antigens [87]. Some studies show that high cholesterol decreases Treg activity [88] and stimulates the presentation of antigens by DCs [89], thereby favoring graft rejection by presenting epitopes from the transplanted kidney.

**Figure 1:**
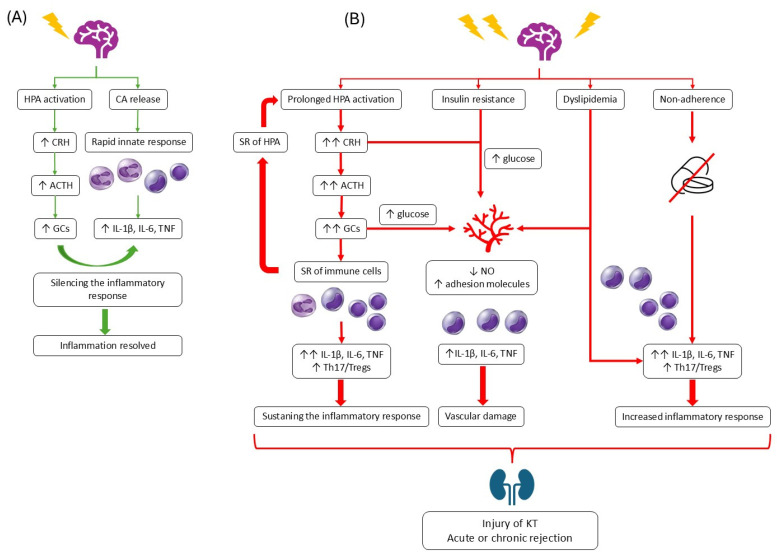
Influence of chronic stress on transplanted kidney. On the right, the response to acute stress is shown (**A**). On the right, the influence of chronic stress on the HPA axis, immune response, vascularity, and adherence is presented (**B**).

Figure 1 summarizes the mechanisms through which chronic stress can influence the fate of KT. All mechanisms described above overlap; for example, some behavioral disorders promote inflammatory processes, and the HPA axis affects not only immune cells but also the hypothalamus and blood vessels, thus sustaining HPA activity, stimulating inflammation, and causing vascular damage.

### 4.5. Clinical Evidence of Stress Influence in KT Patients

Studies show that the quality of KT patients’ lives, the level of anxiety, depression, and stress significantly improve after kidney transplantation. But, in general, patients undergoing KT still face challenges in their physical, psychological, and social adaptation due to lifestyle changes. A study by Jiang et al. [90] demonstrated that KT patients experience moderate stress levels, primarily associated with their financial status. The economic status and factors related to illness and recovery after transplantation seemed to influence overall stress. A study conducted during the COVID-19 pandemic found that 46.2% of KT patients had high perceived stress, as measured by the Perceived Stress Scale (PSS) [91]. 48.1% of them also complained of poor sleep quality. Regression analyses revealed that high perceived stress is an independent predictor of anxiety and depression in that group of patients. In another study, the Transplanted Organ Questionnaire “psychological rejection” subscale was associated with higher serum creatinine (sCr) and estimated glomerular filtration rate (eGFR) levels [92]. Nassar et al. [93], who did not actually examine the effect of stress itself, but rather anxiety and depression, demonstrated that anxiety correlated with elevated sCr in KT recipients.

## 5. Interventions to Reduce the Negative Impact of Stress

Strengthening individuals’ coping skills before problems arise could be beneficial not only for the healthy population but, above all, for populations of somatic patients who, due to struggles with somatic illness, are exposed to additional, often overwhelming stress and sometimes even experience trauma. Supporting their ability to cope with stress could have a positive impact not only on their well-being but, as numerous studies with a psychoneuroimmunological profile show, on their treatment and physical health.

The first step towards better supporting transplant patients is already a part of the transplant procedure. Psychological evaluation is an integral part of the comprehensive assessments patients undergo during the organ transplantation qualification process. Its primary goal is to identify potential maladaptive psychological factors that may pose a risk to the surgical procedure, recovery, and cooperation with physicians (including adherence to their guidelines), thus questioning the success of the entire organ transplant procedure [94]. Psychological evaluation can also have additional applications, such as improving understanding of the patient’s mental functioning, helping medical staff understand the patient’s needs, and determining the level of cognitive functioning, which facilitates adapting medical messages to make them as clear and understandable as possible for the patient. Determining the patient’s social support network is also important in the context of post-operative treatment and in the event of a diagnosis of possible addiction to psychoactive substances. A detailed description of the course of addiction, current stage, treatment implemented, duration of abstinence, and prognosis is closely related to approval for transplantation, especially if addiction was one of the reasons for the need for transplantation, as is the case with patients requiring liver transplantation [95].

The scope of psychological evaluation and the methods used are not standardized and largely depend on the facility performing the evaluation and transplant. However, the expectations of good psychological functioning from a patient undergoing such a serious procedure, associated with a complex post-operative recovery, remain universal. Some centers have also incorporated assessments of the quality of the patient’s social support and interviews with those providing it. This not only provides a picture of the quality of social support in the patient’s life but also often complements the patient’s own functioning, as reflected in observations from the patient and their immediate environment.

Both evaluating the social support system in place for a patient undergoing transplant surgery and inviting loved ones to participate more actively in the treatment process seem to have a positive impact on the entire transplant process. The evaluation is actually the beginning, followed by subsequent stages—such as the waiting period for the organ, a period that is filled with intense stress and psychological discomfort for patients and during which psychological support is often needed; the transplant surgery itself, recovery, and adaptation to the new reality of life with a transplanted organ [95].

At each of these stages, the patient faces strong emotions, increased stress, and tension, which often contribute to the development of mental health disorders. Among transplant patients, the most common challenges include adjustment disorders, depressive and anxiety symptoms, and difficulties with social relationships [94]. Despite numerous studies, there is no clear evidence that these disorders significantly impact post-transplant morbidity and mortality [94]. Psychological support for patients at every stage of transplantation treatment seems to be based more on positive psychology than on the psychology of disorders, with an emphasis on building resilience to stress and on regulating emotions with the support of the environment and the use of the patient’s resources.

### 5.1. Psychotherapy

Psychotherapy’s impact on changes within an individual is often difficult to capture in the way scientists would like. It is rarely focused solely and exclusively on a distinct and independent sphere of the subject’s functioning. This is primarily because in psychology, many spheres of emotional, cognitive, and behavioral functioning intersect. It is very difficult to isolate a single variable in the psychotherapy process and focus solely on it, deliberately avoiding other aspects of the patient’s life.

In their study on the impact of psychological interventions on quality of life and reduced distress in patients awaiting kidney transplantation, Rodrigue et al. [96] demonstrated the challenging and necessary nature of research on the impact of various therapeutic interventions. They randomly introduced three different types of interventions: quality of life therapy (QOLT), supportive therapy (ST) (“ST was chosen as the active treatment alternative because all transplant centers employ social workers who provide educational and supportive intervention”), or standard care (SC), which does not include any psychological intervention. They regularly assessed their impact on measurable indicators such as quality of life, mental functioning, mood, anxiety, depression, and “good” and “bad” days using Quality of Life Inventory (QOLI), SF-36 Health Survey, Profile of Mood States-Short Form (POMS), and Hopkins Symptom Checklist-25 (HSCL). The study showed that QOLT was more effective in improving quality of life than ST. Furthermore, ST and QOLT were found to reduce “bad days” and stabilize patients’ mood. Interestingly, the reduction in distress levels in ST patients did not lead to an improvement in perceived quality of life, unlike in patients receiving QOLT [96]. This study clearly suggests the superior effectiveness of psychotherapy over other methods in supporting patients awaiting kidney transplantation. More research is needed to isolate psychotherapy as a variable clearly responsible for improving the mental state of somatic patients. Studies are also required to demonstrate the impact of physical improvement resulting from the mental improvement provided by psychotherapy. The type of psychological intervention, the therapeutic approach used, and the tools used to work with the patient are also important.

In one meta-analysis of 29 studies including 2668 participants, the effectiveness of Mindfulness-Based Stress Reduction (MBSR), a training focused on reducing stress, depressive symptoms, and anxiety, was examined [97]. MBSR is a complex tool that teaches individuals not only to be present in the moment and to pay attention to the current situation, bodily sensations, and emerging thoughts without analyzing, judging, or reacting to them, but also to acknowledge their existence simply. The meta-analysis states, “MBSR provides training in formal mindfulness practices, including body scan, sitting meditation, and yoga. MBSR seeks to change the individual’s relationship with stressful thoughts and events by decreasing emotional reactivity and enhancing cognitive appraisal.” The results of the meta-analysis suggest that MBSR has a large effect on stress levels, a moderate impact on anxiety, depressive symptoms, distress, and quality of life, and a small effect on occupational burnout. However, the meta-analysis included only healthy individuals, so its results are unlikely to apply to a patient population. Although MBSR is used in groups of patients of various types, the primary interest of researchers seems to be more its impact on the level of depressive and anxiety symptoms than on stress.

One of the few studies showing the effect of MBSR (in a phone-adapted version) on stress levels clearly indicates its effectiveness [97]. MBSR is a method that requires minimal resources and, according to research, can reduce perceived stress. Introducing it as a tool to support somatic patients, especially those whose treatment process can be deeply disrupted by the physiological aspects of stress, such as transplant patients, seems entirely justified.

Almost a decade ago, the positive effects of several weeks of mindfulness-based resilience training (MBRT) on the resilience of transplant patients and their caregivers were also studied [98]. The training was introduced during the pilot phase, and the study and its results highlighted its positive impact on MBRT. The program demonstrated significant improvements across several aspects, including how patients perceived stress. However, it did not receive wider continuation.

### 5.2. Educational and Patient Support Programs

Stress and tension reduction tools, while based on universal mechanisms, can be personalized. In other words, what may be a beneficial form of tension or anxiety reduction for some will exacerbate it for others or may not be as effective. This is due to individual differences in human personalities, patterns and scripts of action and thought, personal life experiences, and many other variables that contribute to our psychological individuality. Therefore, it seems worthwhile not only to present patients with tools for specific actions but also to provide space for feedback and to share it with other patients.

In his article “Life After Kidney Transplantation: The Time for a New Narrative”, Kevin John Fowler, a kidney transplant recipient, highlights an important aspect of shared experiences within the transplant community, writing: “For myself and for many other kidney transplant recipients, we only truly feel understood by fellow recipients. The lack of understanding by some members of the medical community and sometimes our loved ones can create a sense of isolation and loneliness during different parts of our journeys,” pointing to the positive, supportive role a community of fellow recipients can play [99]. He also describes how he coped with the emotions and anxiety he experienced, what tools helped him, and why. He mentions, among other things, physical exercise, which helped him regain control of his life after the transplant, and journaling, which made it easier for him to notice not only the negative aspects of the day but also the positive ones and helped reduce rumination. He describes his exploration of transplantation by reading literature that explains the topic. He also emphasizes the crucial role of the routine he created for himself—a routine that helped him despite numerous health problems after the transplant [99].

One person’s perspective is often lost amid meta-analyses and statistical data. However, it provides important testimony for other patients in the same group. It provides a concrete example of the real usefulness of tools (or those not presented by medical specialists) and reinforces their practicality. After all, shared experience has long been used in support groups, which provide members with a sense of community, understanding, and support. Support groups are highly valued by transplant patients, especially when supported by medical specialists who facilitate recovery [100].

Beyond support, groups can also be educational, building specific skills needed in patients’ daily lives. One such form is an Empowerment Support Group, which, beyond providing support through shared experiences, aims to build a sense of empowerment in participants. “Empowerment involves recognizing, promoting, and enhancing the ability of patients to meet their own needs so that they feel in control of their own lives and their own care” [101]. An Empowerment Support Group brings together patients with the same illness to share experiences, reduce stress, address unspoken anxieties, and alleviate feelings of loneliness. It appears to be training in a sense of control and competence to cope with one’s health situation and related challenges. Studies show a clear, positive impact of such groups on empowerment and self-care among transplant patients in Asia [101].

Research also highlights the value of psychological support in the form of individual contact with a specialist, given individual differences. Psychological support is particularly important in the context of adapting to the changes brought about by transplantation, difficulties in relationships with partners, friends, and family, and the relationship with a graft (“Encourage ways to form a positive relationship, including conceptualizing the relationship as reciprocal or symbiotic. Address issues of possible perceptions of being influenced by the donor”), as well as coping with feelings of uncertainty and risk [102].

### 5.3. Relaxation Techniques

Progressive Muscle Relaxation (PMR), as described by Jacobson, is perhaps one of the best-known relaxation techniques, primarily due to its widespread availability, no cost, no equipment requirements, and the ability to be performed independently, almost anywhere, and without adverse effects [103]. PMR involves deliberately tensing and relaxing muscle groups in response to a speaker’s voice, thereby enabling the isolation of individual muscles or entire muscle groups that are persistently tense. While it may sound unspectacular in theory, PMR is a prime example that not only does the mind affect the body, but also the body affects the mind. The idea is that a relaxed body is less likely to experience psychological discomfort because bottom-up messages from relaxed muscles convey that nothing threatening is happening.

Muscle tension is a fundamental attribute of the fight-or-flight response, and relaxed muscles signal the absence of threat/stress. According to this relationship, PMR should reduce stress and tension levels. Research shows that this is indeed the case. A meta-analysis of over 46 studies involving 3402 adults found PMR to be an effective tool for reducing both stress and symptoms of depression and anxiety in adults. Interestingly, the analysis included studies from 16 countries, demonstrating that, as intended, PMR works unaffected by potential cultural differences because it operates at the level of psychoneurophysiological mechanisms [104].

Thanks to its beauty, simplicity, and efficiency, PMR can be widely used in settings where other activities are precluded (e.g., hospitals). The patient can also continue it after returning home. Research indicates that PMR is not only a popular and effective tool but also has real potential to reduce difficulties in mental functioning [104].

The limited number of studies on the use of PMR to support the well-being of transplant patients suggests its limited widespread use. However, individual studies in transplant patients demonstrate that PMR has been effective in regulating vital signs and reducing fatigue. Uzun Yağız and Avcı [105] conducted an experimental study in which KT patients underwent PMR. The authors used the Descriptive Characteristics Form (DCF), the Vital Signs Monitoring Form (VSMF), and the Fatigue Severity Scale (FSS) to analyze the intervention outcomes. Their results confirmed the effectiveness of PMR in regulating vital signs and reducing fatigue in KT patients.

Among the regulatory methods, we also find various types of breathing exercises. Typically, in terms of their impact on stress and tension levels, they are part of broader interventions, such as meditation, mindfulness practice, or even PMR. As a standalone tool, they do not seem sufficient. A systematic review found that diaphragmatic breathing, when used as an isolated method to reduce mental and physiological stress, is less effective than expected. This may be due to limited research on diaphragmatic breathing as a standalone stress-reducing tool, or it may indicate that diaphragmatic breathing should be part of a more complex activity [106], especially given the unequivocally positive results of complex interventions that include an aspect of conscious breathing.

### 5.4. Preliminary Evidence of the Effectiveness of Stress-Reducing Interventions

Although relatively few, interventional studies provide encouraging evidence that psychological support can improve outcomes in kidney transplant recipients. Randomized and quasi-experimental trials of cognitive-behavioral therapy (CBT), mindfulness-based stress-reducing (MBSR) interventions, and structured social support programs have shown benefits in reducing perceived stress, improving medication adherence, and stabilizing eGFR trajectories. In a study by Gross et al. [107], 8-week MBSR, based on the protocol by Santorelli et al. [108], has been shown to improve the quality of life of recipients of liver, kidney, lung, or heart transplants. The protocol consisted of body scan, sitting meditation, gentle Hatha yoga, and walking meditation. The results were measured with the State-Trait Anxiety Inventory (STAI), the Center for Epidemiological Studies-Depression Scale (CES-D), and the Pittsburgh Sleep Quality Index (PSQI).

But even stable KT recipients can profit from mindfulness techniques. A study by Lim et al. [109] demonstrated that 20 min mindful breathing practiced for 4 weeks can reduce symptoms of anxiety, depression, and insomnia. KT patients who tried this technique reported less fatigue and improved well-being, as measured by the Edmonton Symptom Assessment Scale (ESAS).

### 5.5. Changes in Inflammatory Markers After Psychological Interventions

A meta-analysis by Liang et al. [110] demonstrated that reductions in inflammatory biomarkers (e.g., IL-6, TNF-α) accompany psychological interventions, suggesting potential biological reversibility of stress-induced immune activation. By psychological interventions, the authors meant exposure to music or nature, practicing gratitude, optimism, kindness, and other meaning-focused activities. Analysis revealed that those interventions were successful in reducing pro-inflammatory marker levels but not cortisol levels. The effect was significant in clinical patients from the intensive care unit scheduled for major surgery, cancer patients, patients with Alzheimer’s disease, chronic heart disease or coronary artery disease, or human immunodeficiency virus (HIV). Unfortunately, the analysis did not include patients with CKD or KT.

Another meta-analysis [111] found that the overall effect size across psychological interventions and groups on pro-inflammatory biomarker levels was statistically significant. The analyzed studies included various patients. Among them, one study by Chen et al. [112] reported that 4-week CBT reduced IL-1β levels in patients with ESRD. Simultaneously, the influence of CBT on patients’ well-being was assessed using the PSQI and the Fatigue Severity Scale (FSS).

Despite promising results, most trials remain small and heterogeneous in design, and no studies have examined changes in inflammatory markers after psychological interventions in KT patients. Large-scale, multicenter randomized controlled trials are needed to clarify whether targeted stress management can directly prolong graft survival.

Table 1 summarizes the studies on the effectiveness of different stress-reducing interventions in KT patients.

## 6. Gaps in Knowledge and Direction for Future Research

Despite growing recognition of the role of psychological and physiological stress in kidney transplantation, significant knowledge gaps remain. Current evidence largely derives from small, heterogeneous studies, often limited by cross-sectional designs, subjective stress measures, and a lack of mechanistic integration. Most studies rely on self-reported stress or depression scales without concurrent measurement of objective biomarkers or immune parameters. Longitudinal designs with repeated sampling of both psychological and biological variables are rare. Furthermore, the heterogeneity of transplant populations—in terms of age, comorbidities, immunosuppressive regimens, and socio-economic context—complicates the interpretation and generalization of results.

Although neuroendocrine–immune crosstalk has been proposed as a key mechanism linking stress to graft injury, the specific molecular pathways underlying this crosstalk remain incompletely understood. The roles of individual cytokines (e.g., IL-1β, IL-6, or TNF-α), autonomic imbalance, and glucocorticoid resistance warrant detailed experimental investigation in transplant-specific settings. Emerging omics-based technologies—including transcriptomics, metabolomics, and immunophenotyping—could provide novel insights into how stress modulates immune signatures associated with rejection and fibrosis.

Future research should adopt multidimensional frameworks combining psychological, immunological, and metabolic assessments. Large-scale, prospective cohort studies are needed to establish causality between stress exposure and long-term graft outcomes. Interventional trials testing structured stress-reduction programs (e.g., CBT, mindfulness-based interventions, biofeedback) should include not only psychological endpoints but also biomarkers of inflammation, oxidative stress, and endothelial function. Moreover, personalized approaches integrating psychoneuroimmunological profiling with tailored immunosuppression could open new therapeutic avenues for high-risk patients.

Bridging the gap between basic and clinical research requires better collaboration among transplant physicians, psychologists, and immunologists. Developing standardized protocols for stress screening, biomarker validation, and psychological support within transplant follow-up could markedly enhance patient-centered care and graft longevity.

## 7. Conclusions

Research on post-transplant patients primarily focuses on their physiological functioning, taking into account the potential for complications, the complex recovery process, and the medications they take. In the psychological context, most studies concentrate on depression, anxiety disorders, post-traumatic stress disorder (PTSD), and the broadly understood quality of life. Few, if any, studies examine the impact of stressors unrelated to transplantation on the patient’s psychosocial health. Finding only a few articles showing the relationship between stress and the fate of a diseased or transplanted kidney indicates a significant gap in this field.

Stress represents a multifaceted but underrecognized determinant of kidney graft outcomes. Through complex interactions between the neuroendocrine and immune systems, chronic stress promotes inflammation, endothelial injury, and fibrosis, ultimately jeopardizing long-term graft survival. Clinical evidence supports the association between psychological distress and poorer transplant outcomes, though causality remains to be definitively established. Addressing this interplay requires an integrative biopsychosocial approach that combines psychological assessment, biological monitoring, and supportive interventions as part of routine post-transplant care. Incorporating stress management and mental health support into standard transplant protocols has the potential not only to improve quality of life but also to enhance graft longevity—turning psychoneuroimmunology into a practical component of precision transplantation medicine.

Compared with previously published reviews, our work provides a novel integrative perspective by explicitly articulating the Integrated Stress–Immune–Transplant Kidney Model. While earlier studies have described individual components, such as HPA axis dysregulation, glucocorticoid resistance, inflammatory cytokine shifts, endothelial dysfunction, or psychological determinants of adherence, these have rarely been conceptualized as interconnected elements of a single mechanistic pathway. The model presented here highlights how stress-related neuroendocrine signals converge with immune and vascular processes to shape chronic immune activation and, ultimately, graft outcomes. It also incorporates behavioral and psychological responses as downstream modulators of the same pathway, rather than parallel or unrelated factors. By integrating these domains, the model clarifies causal relationships that remain implicit or fragmented in prior literature and provides a framework to guide both mechanistic research (e.g., linking psychoneuroimmunological measures to protocol biopsy findings) and the development of clinical interventions targeting stress, coping, and behavioral adherence.

## Figures and Tables

**Table 1:** List of studies showing the effectiveness of different stress-reducing interventions.

Author (Year)	Type of Research	Number of Participants	Type of Intervention	Final Results
Gross et al., 2010 [107]	A randomized controlled trial	138 recipients of kidney, kidney/pancreas, liver, heart, or lung transplants	MBSR for 8 weeks	Reduced symptoms of anxiety and depression, improved quality of life
Hsiao et al., 2016 [101]	A randomized controlled trial	122 patients have undergone a renal transplant within the past 20 years	6 meetings with the Empowerment Support Group	Increased levels of empowerment and improved self-care behaviors
Lim et al., 2025 [109]	A randomized controlled trial	60 kidney transplant recipients	20 min mindful breathing for 4 weeks	Greater reduction in the total ESAS score
Rodrigue et al., 2011 [96]	A randomized controlled trial	62 patients awaiting kidney transplantation	6 sessions of quality of life therapy or supportive therapy	QOLT was more effective in improving the quality of life than ST
Stonnington et al., 2016 [98]	A clinical study	31 recipients of heart, liver, kidney/pancreas, and stem cell transplant	MBRT for 6 weeks	Improved quality of life, reduced symptoms of stress, depression, and anxiety
Uzun Yağız and Avcı, 2024 [105]	A randomized controlled trial	52 kidney transplant patients	PMR exercises for 4 weeks	Reduced fatigue

## Data Availability

No new data were created or analyzed in this study. Data sharing is not applicable to this article.

## Abbreviations

The following abbreviations are used in this manuscript:

ABMR	Antibody-mediated rejection
ACTH	Adrenocorticotropic hormone
BBB	Blood–brain barrier
CBT	Cognitive-behavioral therapy
CKD	Chronic kidney disease
CNS	Central nervous system
CRH	Corticotropin-releasing hormone
ESRD	End-stage renal disease
GC	Glucocorticoids
HPA	Hypothalamic–pituitary–adrenal
IL	Interleukin
KT	Kidney transplant
MBSR	Mindfulness-Based Stress Reduction
NO	Nitric oxide
PAIS-SR	Psychosocial Adjustment to Illness Scale Self-Report
PMR	Progressive Muscle Relaxation
PTSD	Post-traumatic stress disorder
PSS	Perceived Stress Scale
QOLT	Quality of life therapy
SR	Steroid resistance
TCMR	T-cell-mediated rejection
TNF	Tumor necrosis factor
